# Evaluation of the visibility of early gastric cancer using linked color imaging and blue laser imaging

**DOI:** 10.1186/s12876-017-0707-5

**Published:** 2017-12-08

**Authors:** Yoshikazu Yoshifuku, Yoji Sanomura, Shiro Oka, Mio Kurihara, Takeshi Mizumoto, Tomohiro Miwata, Yuji Urabe, Toru Hiyama, Shinji Tanaka, Kazuaki Chayama

**Affiliations:** 1 Department of Gastroenterology and Metabolism, Graduate School of Biomedical Sciences, Hiroshima University, Hiroshima, Japan; 2 Department of Endoscopy, Hiroshima University Hospital, 1-2-3 Kasumi, Minami-ku, Hiroshima, 734-8551 Japan; 3 Health Service Center, Hiroshima University, Higashihiroshima, Japan

**Keywords:** Early gastric cancer, Linked color imaging, Blue laser imaging, Visibility

## Abstract

**Background:**

Blue laser imaging (BLI) and linked color imaging (LCI) are the color enhancement features of the LASEREO endoscopic system, which provide a narrow band light observation function and expansion and reduction of the color information, respectively.

**Methods:**

We examined 82 patients with early gastric cancer (EGC) diagnosed between April 2014 and August 2015. Five expert and 5 non-expert endoscopists retrospectively compared images obtained on non-magnifying BLI bright mode (BLI-BRT) and LCI with those obtained via conventional white light imaging (WLI). Interobserver agreement was also assessed.

**Results:**

In experts’ evaluation of the images, an improvement in visibility was observed in 73% (60/82) and 20% (16/82) of cases under LCI and BLI-BRT, respectively. In non-experts’ evaluation of the images, an improvement in visibility was observed in 76.8% (63/82) and 24.3% (20/82) of cases under LCI and BLI-BRT, respectively. There were no significant differences between experts and non-experts in the evaluation of the images. The improvement in visibility was significantly higher with LCI than with BLI-BRT in experts and non-experts (*p* < 0.01). With regard to tumor color on WLI, the improvement in the visibility of reddish and whitish tumors was significantly higher than that of isochromatic tumors when LCI was used. The improvement in visibility with LCI was observed in 71% (12/17) and 74% (48/65) of patients with and without *Helicobacter pylori* (*Hp)* eradication, respectively; no significant difference in improvement was observed between these groups. The interobserver agreement was good to satisfactory at ≥ 0.62.

**Conclusions:**

In conclusion, our study showed that LCI improved the visibility of EGC, regardless of the level of endoscopists’ experience or *Hp* eradication in patients, particularly for EGCs with a reddish or whitish color. The improvement in visibility was significantly higher with LCI than that with BLI.

## Background

Gastric cancer is one of the most common cancers, and is also a common cause of cancer-related death. [1] With the development of endoscopic treatments, such as endoscopic submucosal dissection (ESD), patients with early gastric cancer (EGC) can now undergo curative resection with minimal invasiveness, compared with surgery. [2, 3] Therefore, the detection of the cancer at an early stage is very important to obtain good outcomes. However, it is occasionally difficult to achieve an early diagnosis of patients with EGC using conventional white-light imaging (WLI). Superficial EGCs in particular tend to be missed during conventional gastrointestinal endoscopy. Chromoendoscopy with indigo carmine or acetic acid-indigo carmine mixture offers advantages over conventional gastrointestinal endoscopy [4–9]; however, with this technique the procedure is more complex and time-consuming.

Various image-enhanced endoscopy (IEE) techniques have been developed to enhance microvascular contrast and facilitate the resolution of minute superficial patterns and color differences, including the image processing from the image illuminated by the short wavelength light such as narrow band imaging (NBI) or blue laser imaging (BLI), and post image processing such as flexible spectral imaging color enhancement (FICE) or i-scan. [10–15] These IEE images are simple and easy to obtain, as the procedure only involves pushing a button, without the need for a dye solution.

Fujifilm have developed an endoscopic system, “LASEREO” which quips two lasers as the illumination light source instead of a conventional Xenon lamp. One of them is for the white light illumination by stimulating the phosphor on the tip of the endoscope, and the other one produces short wavelength light for BLI. The BLI light is made from the combination of a strong laser light with a 410 nm wavelength, weak laser light with a 450 nm wavelength, and fluorescent light. The lighting provides three observation modes by changing the intensity of the two lasers: BLI mode, BLI bright mode (BLI-BRT), and white light mode. The BLI-BRT mode is brighter than the BLI mode and is intended to improve the detection of gastrointestinal neoplastic lesions. [15] Recently, linked color imaging (LCI)—a color enhancement feature of the LASEREO system—was developed, which ensures the simultaneous expansion and reduction of color information. By using LCI, a reddish mucosa becomes redder in color, whereas a whitish mucosa becomes whiter in color. Each imaging mode, WLI, BLI, BLI- BRT, LCI, is generated by changing the power balance of these two lasers electronically and the specific digital image processing. Therefore, white light mode, BLI mode, BLI-BRT, and LCI can be easily changed by pushing a button. However, to our knowledge, no detailed reports have been published on the improvement of visibility of EGC using LCI and BLI. If LCI improves the visibility of EGC in this study, the result can provide the bridge between an idea that LCI can be used as a screening tool and the study that reveal the true efficacy of LCI for detecting an EGC in screening endoscopy. In the present study, we aimed to assess the change in visibility of EGC using LCI and BLI compared with WLI.

### Patients and Methods

#### Patients

We enrolled 82 consecutive patients (82 EGCs) who were diagnosed with EGC using LCI and BLI, and who underwent ESD at Hiroshima University Hospital between April 2014 and August 2015. Endoscopic images of the tumor were obtained with WLI, BLI, and LCI. While using BLI, we opted for the BLI-BRT mode to maintain brightness. These images were obtained from the same distance and angle as the non-magnified images.

### Setting of endoscopic equipment

The following equipment was used for the study: WLI, BLI, and LCI endoscopes (EG-L590ZW; FUJIFILM Co, Tokyo, Japan), light sources (LASEREO LL-4450; Fujifilm Co), and video processors (AdvanciaHD VP-4450HD; FUJIFILM Co). In the WLI, the structure enhancement function and color mode were set at H + 2 + 4 and C1. BLI and BLI-BRT as delicate as possible in clinical setting level, not complicated engineering. In the BLI-BRT and LCI, the structure enhancement function and color mode were set at the B8 level and level 1.

### Imaging technique with the LCI system

**Fig. 1:**
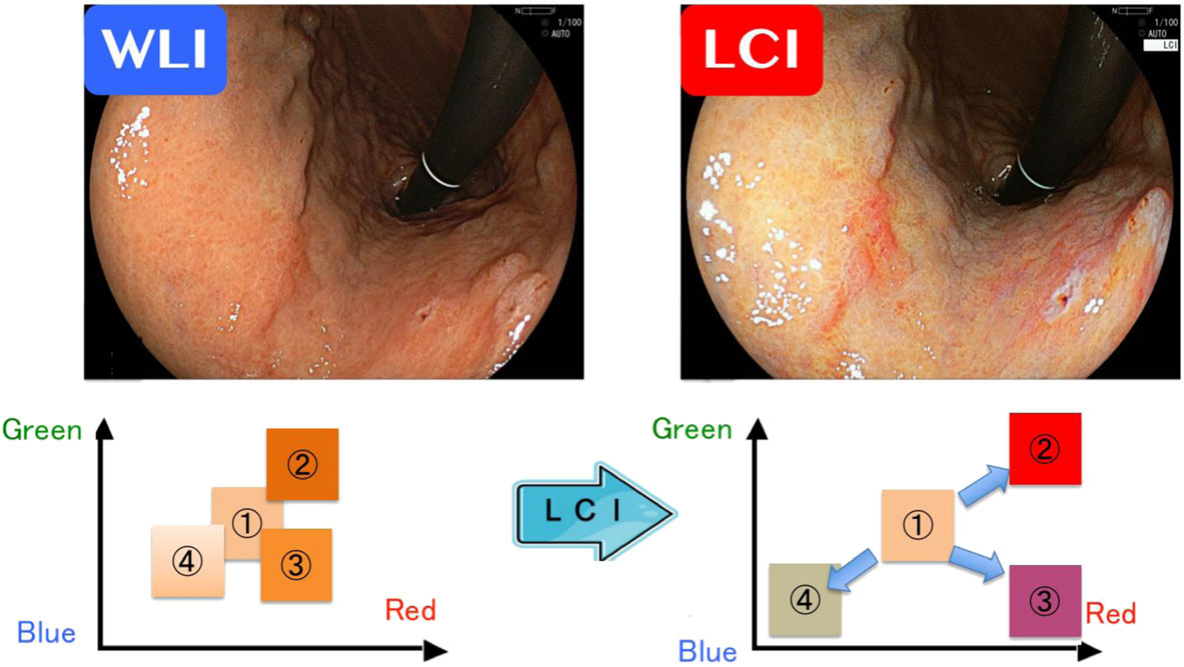
Principle of linked color imaging

In the LCI mode, information of the blood vessels on the mucous surface, the mucosal pattern, and the information acquired by WLI are obtained together by simultaneously irradiating light with a short wavelength (narrow band) and white light in appropriate balance. To easily recognize the differences in colors similar to the mucosal color, the acquired color information is reallocated. Thus, LCI ensures expansion of the color information around the typical mucosal color so that a reddish color appears redder in color, whereas a whitish color appears whiter in color (Fig. 1) However, since LCI keeps the color balance of the blue or green color from conventional white light imaging, the observer doesn’t feel discomfort to the image so much. LCI image can be distinguished the slight color changes on the mucosa such as *Helicobacter pylori* (*Hp*)-associated gastritis or intestinal metaplasia those are sometimes difficult to see by the conventional white light imaging [16–18].

## Methods

**Fig. 2:**
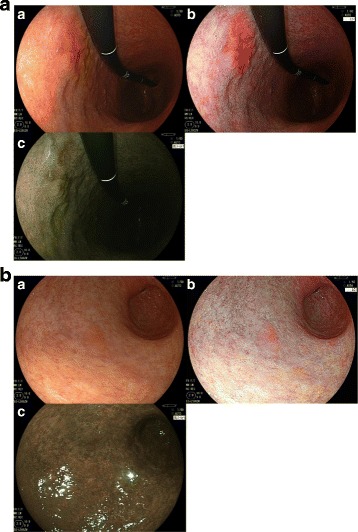
Endoscopic images obtained using linked color imaging (LCI) and blue laser imaging (BLI). A (**a**) White-light imaging (WLI). (**b**) LCI. The experts scored this LCI image +8 points, and classified as improved visibility. The non-experts scored this LCI image +9 points, and classified as improved visibility. (**c**) BLI. The experts scored this BLI image +4 points, and classified as equivalent visibility. The non-experts scored this BLI image +2 points, and classified as equivalent visibility. B (**a**) White-light imaging (WLI). (**b**) LCI. The experts scored this LCI image +5 points, and classified as improved visibility. The non-experts scored this LCI image +7 points, and classified as improved visibility. (**c**) BLI. The experts scored this BLI image −5 points, and classified as worsened visibility. The non-experts scored this BLI image −7 points, and classified as worsened visibility

Five expert endoscopists, who performed > 5000 esophagogastroduodenoscopies, and 5 non-expert endoscopists, who performed ≤ 5000 esophagogastroduodenoscopies, evaluated the images retrospectively. The endoscopic images were presented to each of the endoscopists in a random order for comparison with the images obtained via WLI. Endoscopists scored each of the images obtained via LCI and BLI-BRT for visibility of the tumors according to the following scale: +2 (improved visibility), +1 (somewhat improved visibility), 0 (visibility equivalent to that of WLI), −1 (somewhat worsened visibility), and −2 (worsened visibility), as previously reported. [12] The 10 endoscopists’ scores for each image were tallied. The maximum possible score for any image was +10 and the minimum possible score was −10. A total score of ≥ +5 indicated that the image had an improved visibility, a score between +4 and −4 indicated equivalent visibility, and a score of ≤ −5 indicated worsened visibility (Fig. 2). We also examined the interobserver agreement in relation to the evaluation of the images obtained via LCI and BLI-BRT between the two groups of endoscopists. The interobserver agreement was calculated at 3 levels: improved (+2), equivalent (−1 to +1), and worsened (−2). The patient and tumor characteristics of this study are shown in Table 1.

**Table 1:** Characteristics of the patients and tumors

Factors	No. of patients or tumors (*n* = 82)
Sex ratio (M/F)	53/29
Age, years, mean ± SD	69.6 ± 11.4
Infection with *Hp*	
Positive	65 (79)
Negative	0 (0)
Eradicated	17 (21)
Tumor size, mm, mean ± SD	15.5 ± 11.1
Location of tumor	
Upper	25 (30)
Middle	31 (38)
Lower	26 (32)
Color	
Red	40 (49)
Isochromatic	20 (24)
White	22 (27)
Macroscopic type	
0-IIa	26 (32)
0-IIc	56 (68)
Histological type	
Differentiated	72 (88)
Undifferentiated	10 (12)
Depth	
Mucosa	74 (95)
Submucosa	8 (5)
	(%)

*Hp*, *Helicobacter pylori*

All patients provided written informed consent to undergo ESD and be enrolled in the study. The study was approved by the institutional review board and the ethics committees of Hiroshima University (No. 156). In addition, this study was performed in accordance with the World Medical Association Helsinki Declaration.

### Statistical analysis

Quantitative data are expressed as a mean and standard deviation (SD) or percentages. Rate of improvement between modalities, endoscopists, and clinicopathological features were compared by using the Chi-square test with Yates correction. *P* < 0.05 was considered statistically significant. The interobserver agreement was measured using the kappa statistic. All statistical analysis was performed using JMP software (SAS International Inc., Cary, NC).

## Results

The findings of LCI and BLI-BRT are shown in Table 2. The evaluation of the images by the experts showed that with LCI, improved visibility was observed in 73.1% (60/82) of cases and equivalent visibility was observed in 26.9% (22/82) of cases; none of the cases showed decreased visibility. With BLI-BRT, improved visibility was observed in 19.5% (16/82) of cases, equivalent visibility was observed in 67.0% (55/82) of cases, and worsened visibility was observed in 13.5% (11/82) of cases. The evaluation of the images by the non-experts showed that with LCI, improved visibility was observed in 76.8% (63/82) of cases and equivalent visibility was observed in 23.2% (19/82) of cases; none of the cases showed decreased visibility. With BLI-BRT, improved visibility was observed in 24.3% (20/82) of cases, equivalent visibility was observed in 59.8% (49/82) of cases, and worsened visibility was observed in 15.9% (13/82) of cases. There were no significant differences between experts and non-experts with the evaluation of the images. The improvement in visibility was significantly higher with LCI than that with BLI (*p* < 0.01), and there were no cases where LCI worsened visibility in experts and non-experts.

**Table 2:** Evaluation of the visibility of early gastric cancer with LCI and BLI-BRT, compared to that with white light imaging

Factors	Expert		Non-expert	
	LCI	BLI-BRT	LCI	BLI-BRT
Improved	73.1% (60/82)^a^	19.5% (16/82)^b^	76.8% (63/82)^c^	24.3% (20/82)^d^
Unchanged	26.8% (22/82)	67.0% (55/82)	23.2% (19/82)	59.8% (49/82)
Worsened	0% (0/82)	13.4% (11/82)	0% (0/82)	15.9% (13/82)

*LCI* linked color imaging, *BLI-BRT* blue laser imaging bright mode

a vs b: *p* < 0.01, c vs d: *p* < 0.01

The rate of improvement in visibility using LCI and BLI-BRT according to each clinicopathological feature in experts’ evaluation of images is shown in Table 3. With regard to tumor color using WLI, the rate of improvement in visibility with LCI was 85% (34/40) for reddish tumors, 45% (10/22) for isochromatic tumors, and 80% (16/20) for whitish tumors. The rate of improvement in visibility with LCI was significantly higher for reddish and whitish tumors than for isochromatic tumors. With regard to *Hp* infection, the rates of improvement in visibility with LCI were 74% (48/65) and 71% (12/17) in patients without and with *Hp* eradication, respectively. The improvement in visibility was not significantly different in these groups, regardless of *Hp* eradication. With BLI-BRT, no significant difference in improvement in visibility was observed according to each clinicopathological feature. We also analyzed which clinicopathological features of the lesion had the worse effect on visibility with BLI-BRT; there were no significant differences (Table 4).

**Table 3:** Rate of improvement in visibility of early gastric cancer with LCI and BLI-BRT according to the characteristics of early gastric cancer in experts’ evaluation of the images

Factors	Rate of improvement in visibility	
	LCI	BLI-BRT
Infection with *Hp*		
Positive	74% (48/65)	18% (12/65)
Negative	0% (0/0)	0% (0/0)
Eradicated	71% (12/17)	24% (4/17)
Tumor size, mm		
< 20	70% (40/57)	18% (10/57)
≥ 20	80% (20/25)	24% (6/25)
Location of tumor		
Upper	76% (19/25)	12% (3/25)
Middle	74% (23/31)	23% (7/31)
Lower	69% (18/26)	23% (6/26)
Color		
Red	85% (34/40)^a^	28% (11/40)
Isochromatic	45% (10/22)^b^	18% (4/22)
White	80% (16/20)^c^	5% (1/20)
Macroscopic type		
0-IIa	69% (18/26)	19% (5/26)
0-IIc	75% (42/56)	20% (11/56)
Histological type		
Differentiated	71% (51/72)	19% (14/72)
Undifferentiated	90% (9/10)	20% (2/10)
Depth		
Mucosa	74% (55/74)	22% (16/74)
Submucosa	63% (5/8)	0% (0/8)

*LCI* linked color imaging, *BLI* blue laser imaging bright mode, *Hp Helicobacter pylori*

a vs b, b vs c: *p* < 0.05

**Table 4:** Rate of worse in visibility of early gastric cancer with BLI-BRT according to the characteristics of early gastric cancer in experts’ evaluation of the images

Factors	Rate of worse in visibility with BLI-BRT
Infection with *Hp*	
Positive	11% (7/65)
Negative	0% (0/0)
Eradicated	24% (4/17)
Tumor size, mm	
< 20	16% (9/57)
≥ 20	8% (2/25)
Location of tumor	
Upper	16% (4/25)
Middle	13% (4/31)
Lower	12% (3/26)
Color	
Red	8% (3/40)
Isochromatic	23% (5/22)
White	15% (3/20)
Macroscopic type	
0-IIa	8% (2/26)
0-IIc	16% (9/56)
Histological type	
Differentiated	11% (8/72)
Undifferentiated	30% (3/10)
Depth	
Mucosa	14% (10/74)
Submucosa	13% (1/8)

*LCI* linked color imaging, *BLI-BRT* blue laser imaging bright mode, *Hp*, *Helicobacter pylori*

Interobserver agreements for LCI and BLI-BRT were 0.68 and 0.62 and 0.72 and 0.65 for experts and non-experts, respectively. The interobserver agreement was good to satisfactory, and did not significantly differ between LCI and BLI-BRT (Table 5).

**Table 5:** Interobserver agreements for evaluation of visibility in expert and non-expert

Modality	Interobserver agreement (kappa value)	
	Expert	Non-expert
LCI	0.68	0.72
BLI-BRT	0.62	0.65

*LCI* linked color imaging, *BLI-BRT* blue laser imaging bright mode

## Discussion

In this study, the visibility of EGC with non-magnifying BLI was not significantly improved. By using BLI, the color of the tumor and the surrounding mucosa tend to both be dark because BLI is based on a narrow-band observation function. In the esophagus or colon, we can obtain bright endoscopic images by using BLI-BRT. However, the luminal area of the stomach is much larger than that of the esophagus and colon; hence, this study suggested that non-magnifying BLI may not suitable for detection of EGC. On the other hand, LCI improved the visibility of EGC by 70% or more in experts and non-experts. This result suggests that LCI may be useful in screening or surveillance endoscopy to identify EGC. Moreover, there were no significant differences between the experts and non-experts on the evaluation of images, which suggests that LCI improves the visibility of EGC regardless of the level of the endoscopists’ experience. One major difference between LCI and the other IEE methods is the brightness. For instance, NBI is very reliable and commonly used to diagnose EGC via magnification. [10] However, it is difficult to identify EGCs from a distance as the endoscopic images obtained via NBI are relatively dark. Moreover, meta-analyses have indicated that NBI in conjunction with the previous endoscopy system is not effective for detecting colorectal neoplasia, and that a new generation of NBI devices is needed to improve the brightness. [19, 20] In contrast, LCI images are very bright, and hence, the entire stomach can be clearly observed. Fukuda et al. [18] reported that using LCI, they detected two synchronous flat EGCs that were missed by WLI in a patient who was to undergo ESD for another depressed tumor.

LCI ensures the simultaneous expansion and reduction of color information, and hence, a reddish color would appear to be redder and a whitish color would appear to be whiter; thus, EGC can be more easily detected. Although the visibility of isochromatic tumors did not markedly improve with LCI, as there is only a minor difference in color between these tumors and the surrounding mucosa, most EGCs are reddish or whitish in color, and would be more visible under LCI.

The association between *Hp* infection and gastric cancer development is well established, based on evidence from both epidemiological [21, 22] and experimental studies. [23, 24] Compared to individuals not infected with *Hp*, the odds ratio for gastric cancer is more than 20-fold among individuals infected with *Hp.* [25] A recent prospective study in Japan indicated that the incidence of metachronous gastric cancer is reduced in cases where *Hp* eradication therapy is provided following the endoscopic resection of EGC. [26] Moreover, several studies have indicated that the visibility of EGC after successful *Hp* eradication therapy worsens with WLI as the height of the tumors decrease and the tumor surface becomes unclear due to coverage with low-grade atypia. [27–29] In the present study, we assessed the improvement in the visibility of EGC in cases with and without successful *Hp* eradication therapy. Although no significant difference was observed between these 2 groups, the visibility improvement rate was high even in patients with eradicated *Hp* infection. This result suggests that LCI could improve the visibility of EGC after successful *Hp* eradication therapy.

The present study is the first detailed report that evaluates the visibility of EGC with LCI and BLI. However, this study has some limitations. First, this study is retrospective in nature, and involved a review of endoscopic images. Hence, it may not reflect real-time prospective detection during surveillance endoscopy, and may have some bias. A prospective study should be performed to investigate the detectability of EGC by dividing patients into two groups such that one group is examined by WLI, while the other group is examined by LCI. Second, the study was conducted only in a single academic center in Japan and may lack the generalizability to other practices worldwide.

## Conclusion

In conclusion, our study showed LCI improved the visibility of EGC, regardless of the level of endoscopists’ experience or *Hp* eradication in patients, particularly for EGCs with a reddish or whitish color. The improvement in visibility was significantly higher with LCI than that with BLI.

## Abbreviations

BLI: Blue laser imaging
EGC: Early gastric cancer
ESD: Endoscopic submucosal dissection
FICE: Flexible spectral imaging color enhancement
*Hp*: *Helicobacter pylori*
IEE: Image-enhanced endoscopy
LCI: Linked color imaging
NBI: Narrow band imaging
WLI: White light imaging

## References

[1] Ferlay J, Shin HR, Bray F, Forman D, Mathers C, Parkin DM (2010). Estimates of worldwide burden of cancer in 2008: GLOBOCAN 2008. Int J Cancer.

[2] Tada M, Murakami A, Karita M, Yanai H, Okita K (1993). Endoscopic resection of early gastric cancer. Endoscopy.

[3] Ono H, Kondo H, Gotoda T, Shirao K, Yamaguchi H, Saito D (2001). Endoscopic mucosal resection for treatment of early gastric cancer. Gut.

[4] Ida K, Hashimoto Y, Takeda S, Murakami K, Kawai K (1975). Endoscopic diagnosis of gastric cancer with dye scattering. Am J Gastroenterol.

[5] Yoshinaga S, Gotoda T, Oda I (2009). Clinical imaging of early gastric cancers-conventional endoscopy: including chromoendoscopy using indigo carmine. Stomach Intest (Tokyo).

[6] Iizuka T, Kikuchi D, Hoteya S, Yahagi N (2008). The acetic acid + indigocarmine method in the delineation of gastric cancer. J Gastroenterol Hepatol.

[7] Sakai Y, Eto R, Kasanuki J, Kondo F, Kato K, Arai M (2008). Chromoendoscopy with indigo carmine dye added to acetic acid in the diagnosis of gastric neoplasia: a prospective comparative study. Gastrointest Endosc.

[8] Kawahara Y, Takenaka R, Okada H, Kawano S, Inoue M, Tsuzuki T (2009). Novel chromoendoscopic method using an acetic acid-indigocarmine mixture for diagnostic accuracy in delineating the margin of early gastric cancers. Dig Endosc.

[9] Oka S, Tanaka S, Chayama K, Sanomura Y, Chayama K. Endoscopic diagnosis of early gastric cancer. Nihon Rinsho. 2012;70(10):1742-1747 [Abstract in English]. 23198555

[10] Yao K, Anagnostopoulos GK, Ragunath K (2009). Magnifying endoscopy for diagnosing and delineating early gastric cancer. Endoscopy.

[11] Mouri R, Yoshida S, Chayama K, Oka S, Yoshihara M, Chayama K (2009). Evaluation and validation of computed virtual chromoendoscopy in early gastric cancer. Gastrointest Endosc.

[12] Imagawa H, Tanaka S, Chayama K, Noda I, Higashiyama M, Chayama K (2011). Improved visibility of lesions of the small intestine via capusule endoscopy with computed virtual chromoendoscopy. Gastrointest Endosc.

[13] Osawa H, Yamamoto H, Miura Y, Yoshizawa M, Sunada K, Satoh K (2012). Diagnosis of extent of early gastric cancer using flexible spectral imaging color enhancement. World J Gastrointest Endosc.

[14] Oka S, Tamai N, Ikematsu H, Kawamura T, Sawaya M, Takeuchi Y (2015). Improved visibility of colorectal flat tumors using image-enhanced endoscopy. Dig Endosc.

[15] Yoshida N, Hisabe T, Hirose R, Ogiso K, Inada Y, Konishi H, et al. Improvement in the visibility of colorectal polyps by using blue laser imaging (with video). Gastrointest Endosc 2015;82:542-549. 10.1016/j.gie.2015.01.030 25851158

[16] Dohi O, Yagi N, Onozawa Y, Kimura-Tsuchiya R, Majima A, Itoh Y (2016). Et. Al. linked color imaging improves endoscopic diagnosis of active helicobacter pylori infection. Endosc Int Open.

[17] Ono S, Abiko S, Kato M (2017). Linked color imaging enhances gastric cancer in gastric intestinal metaplasia. Dig Endosc.

[18] Fukuda H, Miura Y, Hayashi Y, Takezawa T, Ino Y, Okada M, et al. Linked color imaging technology facilitates early detection of flat gastric cancers. Clinical. J Gastroenterol. 2015; [Epub ahead of print] 10.1007/s12328-015-0612-9 26560036

[19] Pasha SF, Leighton JA, Das A, Harrison ME, Gurudu SR, Ramirez FC (2012). Comparison of the yield and miss rate of narrow band imaging and white light endoscopy in patients undergoing screening or surveillance colonoscopy: a meta-analysis. Am J Gastroenterol.

[20] Jin XF, Chai TH, Shi JW, Yang XC, Sun QY (2012). Meta-analysis for evaluating the accuracy of endoscopy with narrow band imaging in detecting colorectal adenomas. J. Gastroenterol Hepatol.

[21] Nomura A, Stemmermann GN, Chyou PH, Kato I, Perez-Perez GI, Blaser MJ (1991). Helicobacter pylori infection and gastric carcinoma among Japanese-Ameri- cans in Hawaii. N Engl J Med.

[22] Uemura N, Okamoto S, Yamamoto S, Matsumura N, Yamaguchi S, Yamakido M (2001). Helicobacter pylori infection and the development of gastric cancer. N Engl J Med.

[23] Sugiyama A, Maruta F, Ikeno T, Ishida K, Kawasaki S, Katsuyama T (1998). Helicobacter pylori infection enhances N-methyl-N-nitrosourea-induced stomach carci- nogenesis in the Mongolian gerbil. Cancer Res.

[24] Watanabe T, Tada M, Nagai H, Sasaki S, Nakao M (1998). Helicobacter pylori infection induces gastric cancer in Mongolian gerbils. Gastroenterology.

[25] Ekstrom AM, Maria H, Lars-Erik H, Engstrand L, Nyrén O (2001). Helicobacter pylori in gastric cancer established by CagA immunoblot as a marker of past infection. Gastroenterology.

[26] Fukase K, Kato M, Kikuchi S, Inoue K, Uemura N, Okamoto S (2008). Effect of eradication of helicobacter pylori on incidence of metachronous gastric carcinoma after endoscopic resection of early gastric cancer: an open-label, randomised controlled trial. Lancet.

[27] Ito M, Tanaka S, Takata S, Oka S, Imagawa S, Ueda H (2005). Morphological changes in human gastric tumours after eradication therapy of helicobacter pylori in a short-term follow-up. Aliment Pharmacol Ther.

[28] Matsuo T, Ito M, Takata S, Tanaka S, Yoshihara M, Chayama K (2011). Low prevalence of helicobacter pylori-negative gastric cancer among Japanese. Helicobacter.

[29] Kitamura Y, Ito M, Chayama K, Boda T, Oka S, Yoshihara M (2014). Characteristic epithelium with low-grade atypia appears on the surface of gastric cancer after successful helicobacter pylori eradication therapy. Helicobacter.

